# Vaginal Hysterectomy Versus Vaginal Assisted Natural Orifice Transluminal Endoscopic Surgery Hysterectomy; Results of a Randomised Controlled Trial

**DOI:** 10.1111/1471-0528.70260

**Published:** 2026-05-15

**Authors:** Ilse P. W. Bekkers, Floor R. M. Wintraecken, Nicol A. C. Smeets, Anne Damoiseaux, Jacques W. M. Maas, Huib A. A. M. van Vliet, Martine M. L. H. Wassen

**Affiliations:** ^1^ Department of Gynaecology and Obstetrics Zuyderland Medical Centre Heerlen Heerlen Limburg the Netherlands; ^2^ GROW – Research Institute for Oncology and ReproductionMaastricht University Medical Centre+ Maastricht the Netherlands; ^3^ Department of Gynaecology and Obstetrics Catharina Hospital Eindhoven Eindhoven the Netherlands; ^4^ Department of Obstetrics and Gynaecology Maastricht University Medical Centre+ Maastricht the Netherlands; ^5^ Department of Human Structure and Repair University Gent Gent Belgium

**Keywords:** randomised controlled trial, same‐day discharge, vaginal assisted NOTES hysterectomy, vaginal hysterectomy

## Abstract

**Objective:**

To compare Vaginal Hysterectomy (VH) with Vaginal Assisted Natural Orifice Transluminal Endoscopic Surgery (NOTES) hysterectomy (VANH) as a day‐care procedure.

**Design:**

Single‐blind, multicentre randomised controlled trial.

**Setting:**

Two Dutch non‐academic teaching hospitals.

**Population:**

Women aged ≥ 18 years undergoing hysterectomy for benign indications.

**Methods:**

Women were randomised 1:2 (VH or VANH). Primary outcome was SDD. Secondary outcomes included operative time, rate of elective salpingectomies, intraoperative blood loss, complications (Clavien–Dindo), pain scores (NRS) and analgesic use, post‐operative recovery (RI‐10), and quality of life (EQ‐5D‐5L). Analyses were performed on an intention‐to‐treat basis.

**Results:**

A total of 113 patients were included in the analyses (*n* = 42 VH, and *n* = 71 VANH). SDD occurred significantly more frequently in the VANH group (87.3%) than VH group (71.4%; OR 2.76, 95% CI 1.04–7.25; *p* = 0.04). VANH was associated with a significantly shorter operative time (median 55 min versus 65 min; *p* = 0.005), less blood loss (median 50 mL vs. 150 mL; *p* < 0.001) and more often elective opportunistic salpingectomy compared to VH (100% vs. 77.4%; *p* = 0.008). NRS were significantly lower in the VANH group the first hour post‐operative (3 vs. 1, *p* < 0.001). Post‐operative complications (VH 9.5% vs. VANH 15.5%; *p* = 0.34), readmission (VH 4.8% vs. VANH 8.5%; *p* = 0.47), analgesic use, recovery, and quality of life were not statistically significant.

**Conclusions:**

VANH is a safe and effective alternative to VH, offering a higher likelihood of SDD, shorter operative time, reduced blood loss, and more often an elective salpingectomy, without increased complications or differences in pain, recovery, or quality of life.

## 1 Introduction

Over the past decade, Vaginal Natural Orifice Transluminal Endoscopic Surgery (vNOTES) has been increasingly adopted as a minimally invasive approach for a great variety of benign indications [1, 2, 3, 4, 5, 6]. It combines the benefits of laparoscopic and vaginal surgery, and the global implementation has expanded rapidly supported by growing surgeon experience and scientific evidence.

The first randomised controlled trial (RCT), the HALON trial, comparing hysterectomy by vNOTES with laparoscopic hysterectomy (LH), demonstrated vNOTES to be non‐inferior to LH and allowed more women to be treated in a day‐care setting [7]. vNOTES is associated with shorter operative time, reduced post‐operative pain, and faster recovery compared with LH, while maintaining comparable intra‐ and post‐operative complication rates [7]. A systematic review by Housmans et al. observed a shorter hospital stay and lower analgesic use in the vNOTES group compared to the LH group, without differences in conversion to open surgery or readmission rates [4].

Historically, the Vaginal Hysterectomy (VH) has been considered the preferred surgical route for hysterectomy in women with benign indications, based on its favourable risk–benefit profile and cost‐effectiveness [8, 9, 10, 11, 12]. However, VH faces several challenges. A certain degree of uterine descent is required for safe vaginal access. In addition, performing an opportunistic salpingectomy is more difficult, and this approach does not allow inspection of the abdominal cavity. Moreover, since the introduction of LH and robotic surgery, the skills for VH have decreased [13].

With the emergence of vNOTES, a technique combining the benefits of endoscopic visualization with the established advantages of the vaginal route, important questions arise regarding the current role of conventional VH. Additionally, vNOTES enables performing more complex indications, for example, nulliparity, high BMI, large uterus myomatosus, and multiple caesarean sections [4, 5, 6].

To date, only retrospective studies have compared Vaginal Assisted NOTES Hysterectomy (VANH) with VH, indicating that VANH is a safe alternative with fewer complications and potentially facilitated performance in more complex cases [14, 15, 16]. High‐quality evidence comparing VANH and VH is lacking, highlighting the need for a RCT comparing both techniques in women undergoing hysterectomy for benign indications. This RCT (the VANH trial) has been designed to compare VANH with VH as a day‐care procedure. We hypothesize that women who underwent a VANH can more often be treated in day‐care compared to women who underwent a VH.

## 2 Methods

### 2.1 Study Design and Participants

This study was a single‐blind, multicentre RCT conducted in two non‐university, teaching hospitals in The Netherlands (Zuyderland Medical Centre Heerlen and Catharina Hospital Eindhoven) between July 2021 and September 2025. The study was approved by the ethics board of Zuyderland Medical Centre NL76240.096.21 (METCZ20210035) and was conducted according to the principles of the Declaration of Helsinki. We published the study protocol as an open‐access paper [17].

Inclusion criteria were women aged 18 years and older, Dutch‐speaking, with a benign indication for a VH, and who had given written and oral informed consent. The feasibility of vaginal hysterectomy (VH) was assessed during clinical evaluation by an experienced gynaecologist with expertise in vaginal surgery. Exclusion criteria were a history of more than one caesarean section; (suspected rectovaginal) endometriosis; rectal surgery or pelvic radiation; history of pelvic inflammatory disease (PID); virginity; pregnancy; need for concomitant prolapse or incontinence surgery, or a contraindication for general anaesthesia or preference to stay overnight [18]. Eligible participants were informed about the study and the fact that they were blinded to the allocated intervention.

Randomisation was conducted in a 1:2 ratio, respectively, between the VH and VANH group using the online program ‘Research Manager’ using a permuted block randomisation method with a fixed block of six. A 1:2 randomisation ratio was applied to maximise data collection on vNOTES surgery.

### 2.2 Procedures

The VANH surgeries were performed by four vNOTES trained surgeons: MW, NS, HvV, and AD. The VH's were performed by other gynaecologists experienced in VH in both study centres. All procedures conducted within the study were scheduled to take place before 12 pm.

### 2.3 Interventions

Participants were admitted to the day‐care ward. Preoperative cefazolin 2 g and metronidazole 500 mg were administered intravenously. When feasible, an elective salpingectomy was routinely performed during surgery. Participants received the standard post‐operative pain medication according to the local protocol. The post‐operative pain and discharge protocols in both hospitals were similar.

The post‐operative pain protocol consisted of paracetamol and NSAID's, no opioids were prescribed. The amount of pain medication the patient used per medicine per day was noted by the patients in a journal.

The specific surgical steps for each procedure are described briefly.

#### 2.3.1 VANH

Access to the peritoneal cavity will be performed similar to vaginal surgery by a circular incision around the cervix, anterior and posterior colpotomy and transsecting the sacro‐uterine ligaments. The vNOTES port (GelPOINT V‐Path, Applied Medical) will be placed to get access to the abdominal cavity and a pneumoperitoneum will be created. After positioning in 20° degree Trendelenburg, laparoscopic instruments will be introduced (30° laparoscope, a grasping forceps and sealing device through three trocars). The peritoneal cavity and ureters are inspected. The hysterectomy and (bilateral) salpingectomy are performed by dissecting from caudally to cranially. Finally, haemostasis is checked and the vNOTES port and the uterus (and tubes when dissected) are removed trans‐vaginally and the pneumoperitoneum is deflated. The vaginal cuff will be closed using a running Vicryl‐1 suture.

#### 2.3.2 VH

A circumferential incision is made around the cervix. Access to the peritoneal cavity will be performed through anterior and posterior colpotomy. The hysterectomy and (bilateral) salpingectomy are performed by dissecting from caudally to cranially. The uterus (and tubes when dissected) will be removed and the vagina will be closed. The urinary bladder catheter will be removed directly post‐operative.

### 2.4 Outcome Measurements

The primary outcome was the proportion of SDD in both study groups. Secondary outcomes were surgical outcomes concerning intraoperative complications (Clavien Dindo classification) [19], conversion rate, rate of successful elective salpingectomies, surgery time and blood loss, and post‐operative outcomes concerning complications the first 6 weeks post‐operative (Clavien Dindo classification), post‐operative recovery and quality of life (using the Recovery Index‐10 (RI‐10) at 2 days, 7 days, 4 weeks, 6 weeks and 12 weeks post‐operative and EuroQol 5‐dimensions 5‐levels questionnaire (EQ‐5D‐5L) at six and 12 weeks post‐operative respectively), pain and analgesic use the first 7 days post‐operative (measured using the Numeric Rating Scale [NRS]) [20, 21], costs (using an adapted version of the iMTA Medical Consumption Questionnaire [iMCQ]) after six and 12 weeks post‐operative and cost‐effectiveness. The questionnaires were published in our study protocol [17].

The questionnaire collection was automated. Based on their surgery date, patients automatically received the questionnaires by email after 4, 6, and 12 weeks post‐operative. If they did not fulfil the questionnaire, an automatic reminder would be sent after a couple of days and after 1 week.

### 2.5 Sample Size Calculation

#### 2.5.1 Sample Size

According to literature, we hypothesized a feasibility of 50% SDD in the VH group and 77% in the VANH group, as earlier published in our study protocol [7, 17, 22].

With an alpha of 0.05 and a power of 0.8 and an enrolment ratio of 1 (VH):2 (VANH), this results in a total of 36 patients in the control group and 72 patients in the intervention group. Taking 15% lost to follow‐up into account, we estimated a sample size of 124 patients, of which 41 patients in the VH group and 83 patients in the VANH group.

### 2.6 Statistical Analysis

The data was analysed based on an intention‐to‐treat principle using SPSS (version 29).

No imputation was performed, following the guidelines of Jakobsen et al. [23]. The primary outcome, that is, proportion of SDD between both groups, was analysed by univariable and multivariable logistic regression analyses with adjustments for potential confounders.

For numeric secondary outcomes, the independent sample *t*‐test or Mann–Whitney *U*‐test was used. The categorical secondary outcomes were analysed using univariable and multivariable logistic regression analyses. *p*‐values of < 0.05 were considered to indicate statistical significance.

## 3 Results

Figure S1 showed the CONSORT flow chart of the study. Participant enrolment occurred between June 2021 and September 2025, during which a total of 200 patients were screened for eligibility. A total of 65 patients declined participation. A total of 135 patients were included and randomised to VANH (90 women) and VH (45 women). Twenty‐two women were excluded after inclusion and randomisation due to withdrawal of consent and cancelled surgery at their own request (*n* = 5), pregnancy during the waiting period before surgery (*n* = 1), postponement of surgery beyond the study period (*n* = 1), identifying a malignancy after inclusion (*n* = 2), a wish for concomitant prolapse surgery during the same procedure after inclusion (*n* = 1) and incorrect indication for vaginal surgery identified after inclusion (*n* = 1). An additional 11 patients were excluded due to protocol violations: eight patients were not operated on before 12:00 pm, and three patients were diagnosed intra‐operatively with endometriosis. A total of 19 patients were excluded in the VANH group and 3 patients in the VH group. The primary analysis was performed according to a modified intention‐to‐treat principle, excluding patients with major protocol violations and exclusion criteria as described in our study protocol [17, 24]. A detailed overview of the inclusion and exclusion process can be found in the CONSORT flowchart (Figure S1).

The baseline characteristics are described in Table 1.

**TABLE 1:** Baseline characteristics of the modified intention‐to‐treat principle.

Baseline characteristics	Vaginal hysterectomy (*n* = 42)	Vaginal‐assisted NOTES hysterectomy (*n* = 71)
Race or ethnic group—no (%)		
Caucasian	40 (95)	70 (99)
Asian	0 (0)	1 (1)
Other	1 (2)	0 (0)
Age, years, median (IQR)	42 (38–47)	44 (39–47)
Body mass index, kg/m^2^, median (IQR)	26 (23.4–31.2)	26 (23.4–29.1)
Previous abdominal surgery—no (%)		
None	20 (50)	37 (52)
Laparoscopic surgery	16 (40)	28 (39)
Laparotomic surgery	2 (5)	4 (6)
Endoscopic and laparotomic surgeries	2 (5)	2 (3)
Unknown	2 (5)	0
Parity—no (%)		
Nulliparous	0 (0)	0 (0)
Vaginal delivery	37 (88)	66 (93)
Caesarean section	1 (2)	0 (0)
Caesarean section and vaginal delivery	4 (10)	5 (7)
Indication for hysterectomy—no (%)		
Dysfunctional menstrual bleeding	29 (69)	49 (69)
Cervical dysplasia	2 (5)	4 (6)
Abdominal pain	5 (12)	10 (14)
Post‐ablation/sterilization pain syndrome	3 (7)	2 (3)
Uterine fibroids	3 (7)	5 (7)
Unknown	0 (0)	1 (1)
ASA‐classification—no (%)		
ASA 1	4 (10)	16 (23)
ASA 2	34 (81)	49 (69)
ASA 3	4 (10)	6 (9)
ASA 4	0 (0)	0 (0)
Unknown	0 (0)	0 (0)
Point C under traction, median (IQR)^a^	−2 (−3 to 0)	−1 (−3 to 0.25)
Preoperative Numeric Rating Score, median (IQR)^b^	1 (1–7)	2 (0–6)

*Note:* There were no significant differences (*p* < 0.05) between the two groups in the baseline.

Abbreviations: ASA, America Society of Anesthesiologists; IQR, Inter Quartile Range; NOTES, Natural Orifice Transluminal Endoscopic Surgery; SD, standard deviation.

^a^ 7 values missing in VH group and 7 values missing in VANH group.

^b^ 4 missing values in VH group and 2 missing values in VANH group.

### 3.1 Primary Outcome

The proportion of SDD was significantly higher in the VANH group (87.3%) compared to the VH group (71.4%) (OR 2.76, 95% confidence interval 1.04–7.25; *p* = 0.04) (Table 2).

**TABLE 2:** Perioperative and post‐surgical results.

Outcome	Vaginal hysterectomy (*n* = 42)	Vaginal Assisted NOTES hysterectomy (*n* = 71)	*p*	Effect size (95% CI)
Same day discharge, *n* (%)	30 (71)	62 (87)	0.040	2.76 (1.04–7.25)
Conversion, *n* (%)	1 (2)	1 (1)	0.71	OR 0.59 (0.036–9.62)
Uterine weight in grams, median (IQR)^a^	98 (69–125)	114 (80–154)	0.06	NE
Duration of surgery in minutes, median (IQR)^a^	65 (53–84)	55 (46–69)	0.005	NE
Blood loss in millilitres, median (IQR)^a^, ^b^	150 (100–200)	50 (20–80)	< 0.001	NE
Elective salpingectomy, *n* (%)^c^	24/31 (77)	67/67 (100)	0.008	RR 1.29, 95% CI 1.07–1.56
NRS score, median (IQR)				
1 h post‐operative	3 (2–5)	1 (0–2)	< 0.001	
8 h post‐operative	5 (4–6.8)	4 (3–6)	0.12	
24 h post‐operative morning	6 (4–7)	5 (4–6)	0.21	
Evening	5.5 (3.3–7.8)	5 (3–6)	0.15	
2 days post‐operative morning	4.5 (2.3–6.0)	4 (2–5)	0.07	
Evening	5 (2–7)	3 (2–5)	0.09	
3 days post‐operative morning	3.5 (2–5)	3 (1–4)	0.06	
Evening	3 (2–5)	3 (1–4)	0.24	
4 days post‐operative morning	2 (1–4)	2 (1–3)	0.40	
Evening	3 (1–5)	2 (1–4)	0.19	
5 days post‐operative morning	2 (1–3.8)	2 (1–3)	0.62	
Evening	2 (1–4)	1 (1–3)	0.65	
6 days post‐operative morning	1 (0–3)	1 (1–3)	0.75	
Evening	1 (0–3)	2 (1–3)	0.76	
7 days post‐operative morning	1 (0–3)	1 (0–3.3)	0.66	
Evening	1 (0–3)	1 (0–3.8)	0.43	
Total use analgesics, units, median (IQR)^a^	20 (9–31)	19 (11–27)	0.88	
Complications				
Intraoperative, *n* (%)	0 (0)	1 (1)	0.44	NE
Post‐operative, *n* (%)	4 (10)	11 (16)	0.34	NE
Clavien Dindo grade 1	0 (0)	5 (7)		
Clavien Dindo grade 2	2 (5)	4 (6)		
Clavien Dindo grade 3a/b	2 (5)	2 (3)		
Readmission < 6 weeks, *n* (%)	2 (5)	6 (9)	0.47	OR 1.85 (0.36–9.59)

Abbreviations: IQR, interquartile range; MD, mean difference; NE, not estimable; NOTES, natural orifice transluminal endoscopic surgery; OR, odds ratio; SD, standard deviation.

^a^ Estimated by Mann–Whitney *U*‐test or univariable logistic regression.

^b^ Blood loss was not measured in two patients in the VANH group.

^c^ Salpingectomy was already performed in 11 patients in the VH group and 4 patients in the VANH group.

### 3.2 Secondary Outcomes

#### 3.2.1 Perioperative Outcomes

The VANH group demonstrated a significantly shorter surgical duration compared with the VH group (median 55 min vs. 66 min.; *p* = 0.005) and experienced significantly less intraoperative blood loss (median 50 mL vs. 150 mL; *p* < 0.001).

Elective salpingectomy was significantly more often successfully performed in the VANH group compared to the VH group (100% vs. 77.4%; *p* = 0.008). Logistic regression analysis showed a significantly higher likelihood of successful salpingectomy in the VANH group (RR 1.29, 95% CI 1.07–1.56, *p* = 0.008). Baseline characteristics, including age, BMI, parity, and surgical history, were assessed as potential confounders. No statistically significant differences were observed between groups.

Conversion occurred once in each group. In the VH group, one procedure was converted to VANH because a VH proved difficult due to insufficient uterine descent. In the VANH group, one case was converted to an abdominal hysterectomy due to an intraoperative bladder injury. This patient was 43 years old, had a history of one vaginal delivery and one caesarean section, and a BMI of 19.6 kg/m^2^. This was the only intraoperative complication observed. These differences were not statistically significant (Table 2).

#### 3.2.2 Post‐Operative Outcomes

There were no significant differences in post‐operative complications at 6 weeks following surgery: four patients (9.5%) in the VH group and 11 patients (15.5%) in the VANH group (*p* = 0.34).

In the VH group, the complications included two Grade 2 complications (a vaginal vault hematoma requiring antibiotics and a vaginal vault bleeding) and two Grade 3 complications (severe abdominal pain requiring diagnostic laparoscopy without findings and a vaginal vault hematoma requiring surgical evacuation). In the VANH group, 11 patients (15.5%) experienced post‐operative complications. These included five Grade 1 complications (one arm thrombophlebitis, two vaginal vault hematomas without interventions, one urinary tract infection and one urinary retention), four Grade 2 complications (mixed urinary incontinence, a vaginal vault abscess, readmission due to pain without clinical findings, and a urinary tract infection requiring antibiotic treatment), and two Grade 3 complications (a bladder injury requiring conversion to abdominal hysterectomy and a vaginal vault bleeding requiring surgical re‐intervention).

Readmission rates were 4.8% in the VH group and 8.5% in the VANH group (*p* = 0.47) (Table 2). Three patients were re‐admitted the first day after surgery in the VANH group; all patients experienced too much post‐operative pain. Five patients (*n* = 2 VH group, *n* = 3 VANH group) were admitted due to post‐operative complications.

#### 3.2.3 Pain and Analgesic Use

Pain score was only statistically significantly different in favour of the VANH group at 1 h post‐operatively (Figure S2). Total analgesic use of paracetamol, NSAID's and opioids during the first post‐operative week does not differ significantly between the two groups.

#### 3.2.4 Quality of Life and Post‐Operative Recovery

Quality of life and post‐operative recovery measured respectively using the EQ‐5D‐5L at baseline, 6 and 12 weeks and RI‐10 at 2 and 7 days and 4, 6 and 12 weeks did not differ significantly between the two groups, details are shown in Tables 3 and 4 (for Table 4 see Supporting Information).

**TABLE 3:** Quality of life.

Outcome	Vaginal hysterectomy (*n* = 42)	Vaginal assisted NOTES hysterectomy (*n* = 71)	*p*	OR (95% CI)
Quality of Life (EQ‐5D‐5L)				
QoL baseline (median; IQR)^a^	0.861 (0.75–0.887)	0.861 (0.720–1.000)	0.24	NE
QoL 6 weeks post‐operative (median; IQR)^b^	1.000 (0.848–1.000)	0.887 (0.813–1.000)	0.30	NE
QoL 12 weeks post‐operative (median; IQR)^c^	1.000 (0.884–1.000)	1.000 (0.883–1.000)	0.53	NE

Abbreviations: IQR, inter quartile range, NE, not estimable; NOTES, natural orifice transluminal endoscopic surgery.

^a^ VH 1 missing; VANH 1 missing.

^b^ VH 7 missing; VANH 8 missing.

^c^ VH 10 missing; VANH 12 missing.

## 4 Discussion

### 4.1 Main Findings

The results of this RCT comparing VH and VANH demonstrate that VANH is associated with a significantly higher likelihood of SDD compared with VH. In addition, VANH resulted in significantly less intraoperative blood loss, a shorter operative time, and a higher success rate of opportunistic salpingectomy.

No significant differences were observed concerning conversion rates, intraoperative and post‐operative complications, hospital readmissions, pain scores (except 1 h post‐operative in favour of VANH), use of analgesics during the first week and post‐operative recovery and quality of life up until 12 weeks.

### 4.2 Strengths and Limitations

This study is the first RCT comparing VANH with VH. The multicentre design and the involvement of multiple surgeons enhance the generalizability of the findings to various clinical settings. Furthermore, the use of a standardised surgical protocol described in the published study protocol increased the external validity of the findings. Additionally, patients were blinded to the allocated surgery technique [17].

Since the introduction and implementation of the LH, a decrease in vaginal hysterectomies has been observed [25, 26]. The implementation of vNOTES has the potential to increase the utilisation of vaginal surgical approaches by offering a feasible alternative to laparoscopic procedures, including LH.

The study had several limitations. Several patients were excluded after randomisation due to the unexpected existence of an exclusion criterion, for example endometriosis and pregnancy, and protocol violations. Notably, these exclusions were not balanced between the two groups, with 19 patients excluded from the VANH group and 3 from the VH group. This imbalance could potentially affect the internal validity of the group comparisons.

Another limitation of this study is that the Pelvic Organ Prolapse Quantification (POP‐Q) system was not comprehensively assessed, as only point C was recorded. Since the study population consisted of women undergoing hysterectomy for a variety of benign indications rather than patients with primary urogynaecology complaints, a full POP‐Q evaluation was not considered essential for patient selection. However, the absence of complete POP‐Q data may limit the generalizability of our findings.

### 4.3 Interpretation (In Light of Other Evidence)

The rate of SDD observed in this study was high in both groups, with 87.3% in the VANH group and 71.4% in the VH group. These proportions exceed those reported in previous studies, where SDD rates of approximately 77% following VANH and 63% following VH have been described [7, 22]. This might be explained by the preoperative counselling at the outpatient clinic, in which patients were explicitly prepared for SDD. Preoperative expectation management has been shown to influence post‐operative recovery and discharge readiness; patients who anticipate SDD may be more motivated and better prepared to meet discharge criteria than those expecting an overnight hospital stay [27, 28, 29].

We hypothesized higher SDD rates in the VANH group due to less post‐operative pain and thereby quicker recovery, but the post‐operative pain scores at 8 h did not significantly differ between the two groups. However, in the VANH group there is a trend in lower pain scores compared to the VH group during the first 3 days post‐operative which might be clinically relevant.

In this study, we chose the conventionally performed VH, with no electronic coagulation device use. A RCT comparing electrosurgical bipolar vessel sealing versus conventional clamping and suturing for VH showed shorter surgical time when using a vessel sealing device with a lower pain score the first evening after surgery, but blood loss and hospital stay did not differ [30].

Our study showed a significantly higher percentage of elective salpingectomy. According to literature, an elective salpingectomy during hysterectomy for benign indications does not result in different surgical outcomes, but does lower the risk of ovarian cancer [31, 32].

The conversion rate for VANH observed in this study (1.4%) is consistent with previously published data, including the cohort study by Stuart et al., which reported a conversion rate of 1.6% [33]. Similarly, the conversion rate for VH in the present cohort (2.4%) falls within the range reported in the literature (2.5%–7.5%) [34].

The intraoperative complication rate associated with VANH was low (1.4%) and compares favourably with earlier reports, including the HALON trial (3%) and the study by Stuart et al. (3.2%) [7, 33]. In contrast, the post‐operative complication rate following VANH was higher than that reported in these studies (15.5% vs. 9% in the HALON trial and 2.5% in the cohort study by Stuart et al.) [7, 33]. This difference may be partly attributable to under reporting of post‐operative complication in the cohort study of Stuart et al.; such as cystitis (0.4%), vaginal vault hematoma (0.2%) and post‐operative urine retention (0.07%) [33]. In other studies on vNOTES and hysterectomy post‐operative urinary tract infection rate ranged between 9% and 13% [35, 36] and post‐operative vaginal vault hematoma as a complication after hysterectomy was even observed in 14.6% of the patients [36]. Notably, the post‐operative complication rate following VH in the present study (9.5%) was slightly lower than that reported in the literature (11.6%), suggesting comparable or favourable post‐operative safety outcomes for conventional VH within our RCT [37].

An economic evaluation was performed alongside the clinical trial to determine the cost‐effectiveness of VANH compared to VH. The detailed methodology and results of this analysis will be presented in a separate publication.

## 5 Conclusions

This first single‐blind, multicentre RCT comparing VANH with VH demonstrates a significantly higher rate of SDD in the VANH group, along with several peri‐operative advantages. VANH was associated with shorter operative time, reduced blood loss and a higher likelihood of successful elective salpingectomy, without an increase in complications and with comparable post‐operative recovery. These findings suggest that VANH may represent a valuable alternative to VH for benign indications.

## Author Contributions

**Ilse P. W. Bekkers:** writing – original draft, project administration, validation, project administration, methodology, investigation, formal analysis, data curation, conceptualisation. **Floor R. M. Wintraecken:** writing – original draft, formal analysis, data curation. **Martine M. L. H. Wassen:** writing – review and editing, writing – original draft, supervision. **Nicol A. C. Smeets:** review and editing. **Huib A. A. M. van Vliet:** review and editing. **Anne Damoiseaux:** review and editing. **Jacques W. M. Maas:** review and editing.

## Funding

The authors have nothing to report.

## Ethics Statement

Zuyderland Medical Centre NL76240.096.21 (METCZ20210035), date: 27‐05‐2021.

## Consent

This study involved human subjects, and informed consent was obtained from all participants.

## Conflicts of Interest

Anne Damoiseaux: Payment or honoraria for lectures, presentations, speakers bureaus, manuscript writing or educational events = Applied Medical (Tutor vNOTES surgery) and Coloplast (Tutor ALTIS incontinence surgery). Martine M. L. H. Wassen: Payment or honoraria for lectures, presentations, speakers bureaus, manuscript writing or educational events = Applied Medical (Lectures and educational activities facilitated byApplied Medical. The payments are donated to the research fund of department of Obstetrics and Gynecology of the Zuyderland Medical Centre). Jacques W. M. Maas: Payment or honoraria for lectures, presentations, speakers bureaus, manuscript writing or educational events = Olympus (Trainer GET UP course, payment to my institution) and Leadership or fiduciary role in other board, society, committee or advocacy group, paid or unpaid; Board member World Endometriosis Society and Member SIG endometriosis ESGE. Huib A. A. M. van Vliet: Payment or honoraria for lectures, presentations, speakers bureaus, manuscript writing or educational events = Applied Medical (HAAM van Vliet received fees from Applied Medical on an hourly basis for lectures/educational events on vNOTES. All the fees were donated to a foundation which promotes research in obstetrics and gynecology). The other authors declare no conflicts of interest.

## Supporting information


**Figure S1:** Study flow diagram.

**Figure S2:** Post‐operative Numeric Rating Scores with ranges.*p < 0.001. M, Morning, E, Evening; NRS, Numeric Rating Score; NOTES, Natural Orifice Transluminal Endoscopic Surgery. See Table 2 for the standard deviations and *p*‐values.

**Data S3:** Supporting Information.

## Data Availability

The data that support the findings of this study are available from the corresponding author upon reasonable request.
