# Supplementary material for: Vaginal Hysterectomy Versus Vaginal Assisted Natural Orifice Transluminal Endoscopic Surgery Hysterectomy; Results of a Randomised Controlled Trial
Source: BJOG. 2026 May 15;133(11):2035–43. doi: 10.1111/1471-0528.70260 (PMC13571889; doi:10.1111/1471-0528.70260)
Supplement: Supplementary file 1 [file BJO-133-2035-s001.docx]

**Supplements**

Table 4. *Recovery post-operative*

Ilse Bekkers

Vaginal hysterectomy versus Vaginal Assisted Natural Orifice Transluminal Endoscopic Surgery hysterectomy; results of a randomised controlled trial

Click or tap here to enter text.

| **Time frame: past 36 months** | | |
| --- | --- | --- |
| **2** | Grants or contracts from any entity (if not indicated in item #1 above). |  |
| **3** |  |  |
| **4** | Consulting fees |  |
| **5** |  |  |
| **6** | Payment for expert testimony |  |
| **7** | Support for attending meetings and/or travel |  |
| **8** | Patents planned, issued or pending |  |
| **9** | Participation on a Data Safety Monitoring Board or Advisory Board |  |
| **10** | Leadership or fiduciary role in other board, society, committee or advocacy group, paid or unpaid |  |
| **11** | Stock or stock options |  |
| **12** | Receipt of equipment, materials, drugs, medical writing, gifts or other services |  |
| **13** | Other financial or non-financial interests |  |
|  |  |  |
| **Please place an “X” next to the following statement to indicate your agreement:** | | |
| ☒ | I certify that I have answered every question and have not altered the wording of any of the questions on this form. | |

| Baseline characteristics | Vaginal hysterectomy (n = 42) | Vaginal assisted NOTES hysterectomy (n = 71) |
| --- | --- | --- |
| Race or ethnic group - no (%) |  |  |
| Caucasian | 40 (95) | 70 (99) |
| Asian | 0 (0) | 1 (1) |
| Other | 1 (2) | 0 (0) |
| Age, years, median (IQR) | 42 (38-47) | 44 (39-47) |
| Body Mass Index, kg/m^2^, median (IQR) | 26 (23.4-31.2) | 26 (23.4-29.1) |
| Previous abdominal surgery - no (%) |  |  |
| None | 20 (50) | 37 (52) |
| Laparoscopic surgery | 16 (40) | 28 (39) |
| Laparotomic surgery | 2 (5) | 4 (6) |
| Endoscopic and laparotomic surgeries | 2 (5) | 2 (3) |
| Unknown | 2 (5) | 0 |
| Parity - no (%) |  |  |
| Nulliparous | 0 (0) | 0 (0) |
| Vaginal delivery | 37 (88) | 66 (93) |
| Caesarean section | 1 (2) | 0 (0) |
| Caesarean section and vaginal delivery | 4 (10) | 5 (7) |
| Indication for hysterectomy – no (%) |  |  |
| Dysfunctional menstrual bleeding | 29 (69) | 49 (69) |
| Cervical dysplasia | 2 (5) | 4 (6) |
| Abdominal pain | 5 (12) | 10 (14) |
| Post ablation/sterilization pain syndrome | 3 (7) | 2 (3) |
| Uterine fibroids | 3 (7) | 5 (7) |
| Unknown | 0 (0) | 1 (1) |
| ASA-classification - no (%) |  |  |
| ASA 1 | 4 (10) | 16 (23) |
| ASA 2 | 34 (81) | 49 (69) |
| ASA 3 | 4 (10) | 6 (9) |
| ASA 4 | 0 (0) | 0 (0) |
| Unknown | 0 (0) | 0 (0) |
| Point C under traction, median (IQR)* | -2 (-3 - 0) | -1 (-3 - -0.25) |
| Preoperative Numeric Rating Score, median (IQR)** | 1 (1-7) | 2 (0-6) |

There were no significant differences (p<0.05) between the two groups in the baseline

* 7 values missing in VH group and 7 values missing in VANH group

** 4 missing values in VH group and 2 missing values in VANH group

NOTES = Natural Orifice Transluminal Endoscopic Surgery; ASA = America Society of Anesthesiologists; SD = standard deviation; IQR = Inter Quartile Range

Table 1. *Baseline characteristics of the modified intention-to-treat principle.*

| Outcome | Vaginal hysterectomy (n=42) | Vaginal Assisted NOTES hysterectomy (n=71 ) | p-value | Effect size (95% CI) |
| --- | --- | --- | --- | --- |
| Same day discharge, n (%) | 30 (71) | 62 (87) | 0.040 | 2.76 (1.04-7.25) |
| Conversion, n (%) | 1 (2) | 1 (1) | 0.71 | OR 0.59 (0.036-9.62) |
| Uterine weight in grams, median (IQR) * | 98 (69-125) | 114 (80-154) | 0.06 | NE |
| Duration of surgery in minutes, median (IQR) * | 65 (53-84) | 55 (46-69) | 0.005 | NE |
| Blood loss in millilitres, median (IQR) *. ** | 150 (100-200) | 50 (20-80) | <0.001 | NE |
| Elective salpingectomy, n (%) *** | 24/31 (77) | 67/67 (100) | 0.008 | RR 1.29, 95% CI 1.07-1.56 |
|  |  |  |  |  |
| NRS score, median (IQR) |  |  |  |  |
| One hour postoperative | 3 (2-5) | 1 (0-2) | <0.001 |  |
| Eight hours postoperative | 5 (4-6.8) | 4 (3-6) | 0.12 |  |
| 24 hours postoperative morning | 6 (4-7) | 5 (4-6) | 0.21 |  |
| evening | 5.5 (3.3-7.8) | 5 (3-6) | 0.15 |  |
| 2 days postoperative morning | 4.5 (2.3-6.0) | 4 (2-5) | 0.07 |  |
| evening | 5 (2-7) | 3 (2-5) | 0.09 |  |
| 3 days postoperative morning | 3.5 (2-5) | 3 (1-4) | 0.06 |  |
| evening | 3 (2-5) | 3 (1-4) | 0.24 |  |
| 4 days postoperative morning | 2 (1-4) | 2 (1-3) | 0.40 |  |
| evening | 3 (1-5) | 2 (1-4) | 0.19 |  |
| 5 days postoperative morning | 2 (1-3.8) | 2 (1-3) | 0.62 |  |
| evening | 2 (1-4) | 1 (1-3) | 0.65 |  |
| 6 days postoperative morning | 1 (0-3) | 1 (1-3) | 0.75 |  |
| evening | 1 (0-3) | 2 (1-3) | 0.76 |  |
| 7 days postoperative morning | 1 (0-3) | 1 (0-3.3) | 0.66 |  |
| evening | 1 (0-3) | 1 (0-3.8) | 0.43 |  |
|  |  |  |  |  |
| Total use analgesics, units, median (IQR)* | 20 (9-31) | 19 (11-27) | 0.88 |  |
| Complications |  |  |  |  |
| Intraoperative, n (%) | 0 (0) | 1 (1) | 0.44 | NE |
| Postoperative, n (%) | 4 (10) | 11 (16) | 0.34 | NE |
| *Clavien Dindo grade 1* | 0 (0) | 5 (7) |  |  |
| *Clavien Dindo grade 2* | 2 (5) | 4 (6) |  |  |
| *Clavien Dindo grade 3a/b* | 2 (5) | 2 (3) |  |  |
| Readmission < 6 weeks, n (%) | 2 (5) | 6 (9) | 0.47 | OR 1.85 (0.36-9.59) |

NOTES = Natural Orifice Transluminal Endoscopic Surgery; OR = odds ratio, MD = mean difference, SD = standard deviation, IQR = interquartile range, NE = Not estimable

* Estimated by Mann-Whitney U-test or univariable logistic regression

** Blood loss was not measured in two patients in the VANH group

*** Salpingectomy was already performed in 11 patients in the VH group and 4 patients in the VANH group

Table 2. *Perioperative and postsurgical results*

| Table 3. Quality of Life |  |  |  |  |
| --- | --- | --- | --- | --- |
| Outcome | **Vaginal hysterectomy (n = 42)** | **Vaginal assisted NOTES hysterectomy (n = 71)** | **p-value** | **OR (95% CI)** |
| Quality of Life (EQ-5D-5L) |  |  |  |  |
| QoL baseline (median; IQR)* | 0.861 (0.75-0.887) | 0.861 (0.720-1.000) | 0.24 | NE |
| QoL 6 weeks post-operative (median; IQR)** | 1.000 (0.848-1.000) | 0.887 (0.813-1.000) | 0.30 | NE |
| QoL 12 weeks post-operative (median; IQR)*** | 1.000 (0.884-1.000) | 1.000 (0.883-1.000) | 0.53 | NE |

NOTES = Natural Orifice Transluminal Endoscopic Surgery, IQR = Inter Quartile Range, NE = Not estimable

* VH 1 missing; VANH 1 missing

** VH 7 missing; VANH 8 missing

*** VH 10 missing; VANH 12 missing

Table 3. *Quality of Life*

| Table 4. Recovery post-operative |  |  |  |  |
| --- | --- | --- | --- | --- |
| Recovery Index - 10 (RI-10) | **VH (n = 42)** | **VANH (n = 71)** | **p-value** | **OR (95% CI)** |
| RI-10 2 days post-operative (median, IQR)* | 27.5 (18-32.3) | 30 (23-33.8) | 0.088 | Not estimable |
| RI-10 7 days post-operative (mean; SD)** | 31.26 (7.3) | 32.27 (7.93) | 0.546 | MD -1.006 (-4.305-2.292) |
| RI-10 4 weeks post-operative (median, IQR) *** | 37 (32-41) | 39 (32.5-43) | 0.226 | Not estimable |
| RI-10 6 weeks post-operative (mean; SD)**** | 37.58 (8.79) | 39.32 (7.59) | 0.329 | MD -1.736 (-5.252-1.781) |
| RI-10 12 weeks post-operative (median, IQR)***** | 45 (35.5-48) | 45 (36.3-49) | 0.691 | Not estimable |

* VH 10 missing, VANH 15 missing

** VH 8 missing, VANH 12 missing

*** VH 7 missing, VANH 14 missing

**** VH 11 missing, VANH 11 missing

***** VH 9 missing, VANH 15 missing

Table 4. Recovery post-operative
